# Development and validation of a novel epigenetic-related prognostic signature and candidate drugs for patients with lung adenocarcinoma

**DOI:** 10.18632/aging.203315

**Published:** 2021-07-20

**Authors:** Zhihao Wang, Kidane Siele Embaye, Qing Yang, Lingzhi Qin, Chao Zhang, Liwei Liu, Xiaoqian Zhan, Fengdi Zhang, Xi Wang, Shenghui Qin

**Affiliations:** 1 Institute of Pathology, Tongji Hospital, Tongji Medical College, Huazhong University of Science and Technology, Wuhan 430030, China; 2 Department of Pharmacy, Hiser Medical Center of Qingdao, Qingdao 266033, China; 3 Department of Pathology, Wuhan Third Hospital, Tongren Hospital of Wuhan University, Wuhan 430030, China

**Keywords:** lung adenocarcinoma, epigenetic-related genes, drugs, prognostic, TCGA

## Abstract

Background: Epigenetic dysregulation has been increasingly proposed as a hallmark of cancer. Here, the aim of this study is to establish an epigenetic-related signature for predicting the prognosis of lung adenocarcinoma (LUAD) patients.

Results: Five epigenetic-related genes (ERGs) (ARRB1, PARP1, PKM, TFDP1, and YWHAZ) were identified as prognostic hub genes and used to establish a prognostic signature. According our risk score system, LUAD patients were stratified into high and low risk groups, and patients in the high risk group had a worse prognosis. ROC analysis indicated that the signature was precise in predicting the prognosis. A new nomogram was constructed based on the five hub genes, which can predict the OS of every LUAD patients. The calibration curves showed that the nomogram had better accuracy in prediction. Finally, candidate drugs that aimed at hub ERGs were identified, which included 47 compounds.

Conclusions: Our epigenetic-related signature nomogram can effectively and reliably predict OS of LUAD patients, also we provide precise targeted chemotherapeutic drugs.

Methods: The genomic data and clinical data of LUAD cohort were downloaded from the TCGA database and ERGs were obtained from the EpiFactors database. GSE31210 and GSE50081 microarray datasets were included as independent external datasets. Univariate Cox, LASSO regression, and multivariate Cox analyses were applied to construct the epigenetic-related signature.

## INTRODUCTION

Lung cancer, which including two main types small cell lung cancer (SCLC) and non–small cell lung cancer (NSCLC), is the most common cause of cancer deaths in the world [1]. NSCLC accounts for ~85% of lung cancer diagnoses and imposes a heavy burden on global health systems [2, 3]. Lung adenocarcinoma (LUAD) is the most common pathological subtype of NSCLC. Despite great advances in the treatment of LUAD, the clinical outcome is not satisfactory [4]. Recently, a number of molecularly targeted therapies have been developed that caused significant improvement in the treatment of LUAD, especially for patients with EGFR mutation [5] and ALK mutation [6, 7]. However, because of high gene mutation heterogeneity and complexity in molecular patterns of LUAD, large amount of LUAD patients without EGFR and ALK mutations lose the opportunity to use effective therapeutic drugs. Therefore, it is essential to identify reliable diagnostic and prognostic biomarkers for LUAD patients and thereby provide precise targeted therapies.

Epigenetic regulation is broadly defined as repression or activation of gene expression via DNA methylation and histone modification, without introducing changes in the DNA sequence per se [8, 9]. Epigenetic dysregulation is considered as an essential hallmark in the initiation and progression of different types of cancers [10, 11]. Recently, epigenetic dysregulation has been reported to promote carcinogenesis of pulmonary epithelial cells and the progression of LUAD [12]. Epigenetic genes, such as DNMT, HDAC, and PARP1 have emerged as attractive targets for the development of anticancer drugs for NSCLC [13–15]. Hence, epigenetic alterations are considered to be an important characteristic in NSCLC development and progression. Although, numerous studies have reported prognostic signature for LUAD [16–19], only a few researchers have explored the role of epigenetic prognostic markers in LUAD and found out potential drug candidates for targeted therapy.

In the present study, we firstly developed an epigenetic-related prognostic signature based on TCGA dataset of 497 LUAD patients, and then validated it in both GSE31210 and GSE50081 datasets. More importantly, a new nomogram was created for predicting the overall survival (OS) of LUAD patients based on five epigenetic-related genes (ERGs). The accurate prediction function of the nomogram was evaluated. Moreover, we further screened out candidate targeted chemotherapy drugs to the five ERGs. In conclusion, our study may provide new ideas for epigenetic-related prognostic biomarkers, thereby, highlighting the need to identify high risk patients and eventually delivering more effective targeted chemotherapeutic drugs for LUAD patients.

## RESULTS

### Identifying differentially expressed ERGs

**Figure 1:**
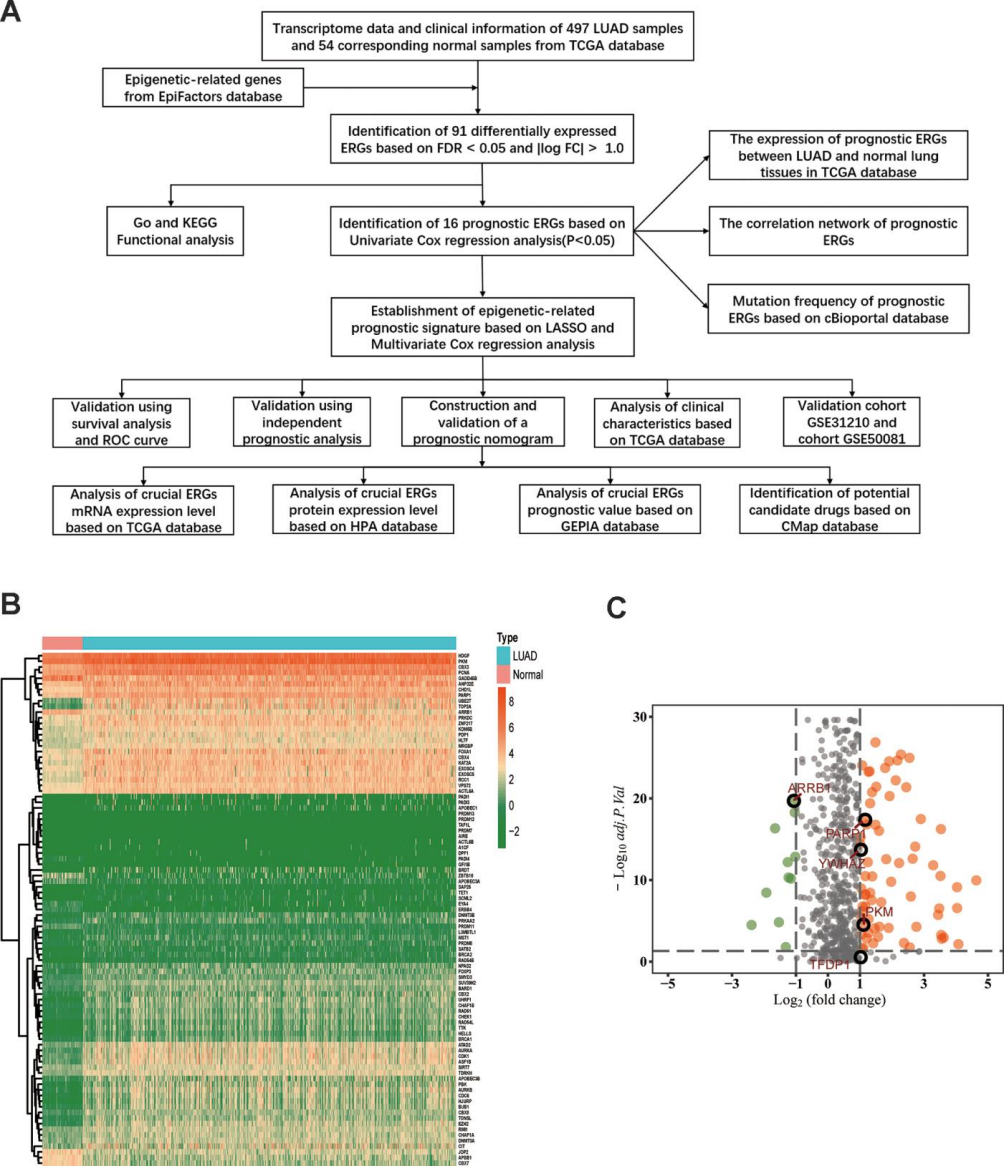
**Differentially expressed epigenetic-related genes (ERGs) in lung adenocarcinoma (LUAD).** (**A**) A flow diagram of the study. (**B**) Heatmap of ERGs between LUAD and nontumor tissues in TCGA database. The color from green to orange represents the progression from low expression to high expression. (**C**) Volcano plot of ERGs in TCGA database. The orange dots in the plot represents upregulated genes and green dots represents downregulated genes with statistical significance. Gray dots represent no differentially expressed genes.

The flow diagram of our study is illustrated in Figure 1A. Firstly, we downloaded mRNA data of 497 LUAD samples and 54 corresponding normal lung samples and corresponding clinical information from the TCGA database. Meanwhile, a total of 720 ERGs were obtained from the EpiFactors database. Then, we matched 720 ERGs with LUAD-related mRNA data, 91 differentially expressed ERGs were identified (|log FC| > 1.0, adjusted *P* value < 0.05), including 12 downregulated and 79 upregulated ERGs (Figure 1).

### GO and KEGG enrichment analyses

**Figure 2:**
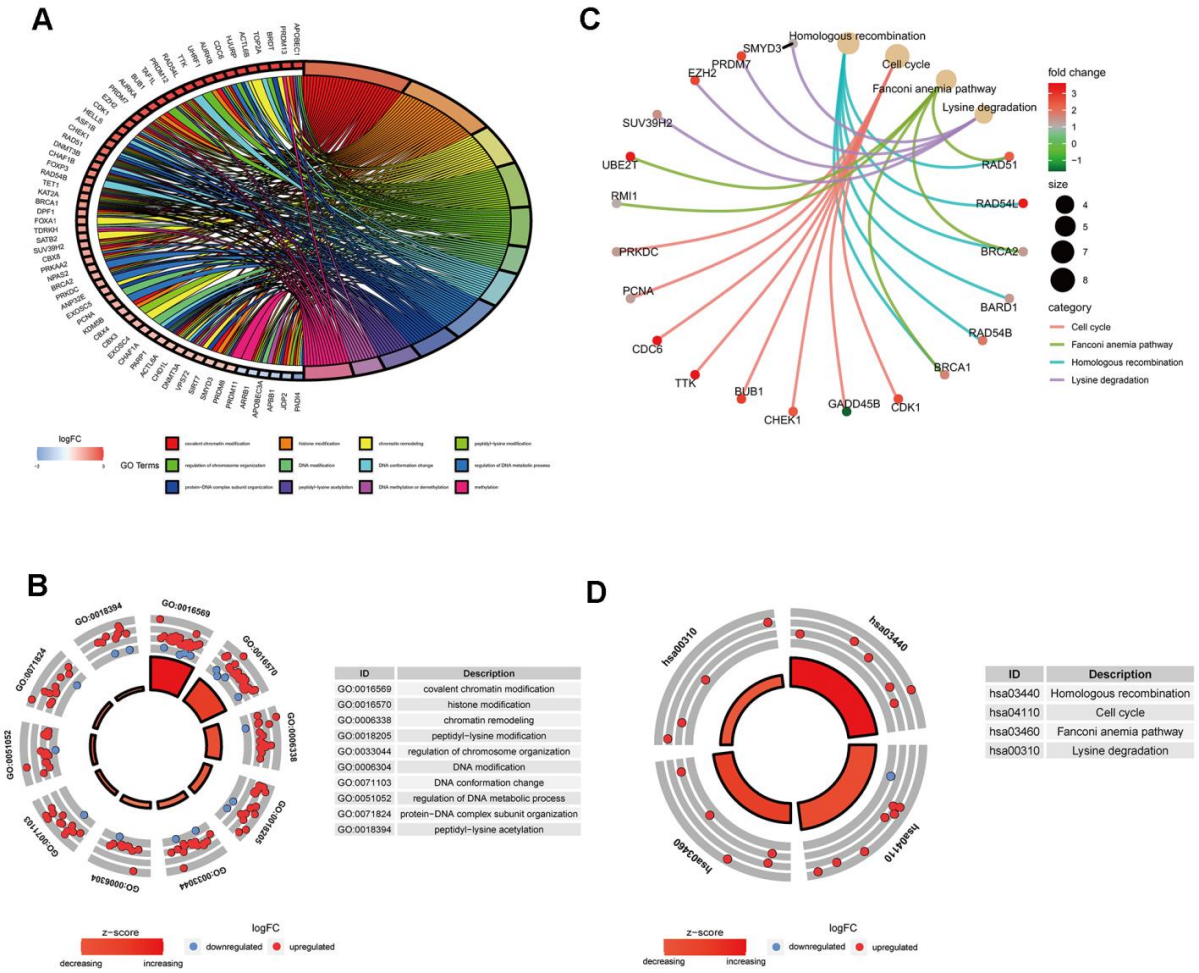
**Gene functional enrichment analysis of differentially expressed ERGs.** (**A**) The top 12 significant terms of GO function enrichment. (**B**) The GO circle shows the scatter map of the logFC of the specified gene. (**C**) The correlation between intersection genes and the significant terms of KEGG. (**D**) The KEGG circle shows the scatter map of the logFC of the specified gene. The higher the Z-score value indicated, the higher expression of the enriched pathway.

GO analysis results indicated that 91 ERGs were mainly enriched in 422 Go terms, such as covalent chromatin modification, histone modification, chromatin remodeling, peptidyl-lysine modification, regulation of chromosome organization and so forth (Figure 2A, 2B). KEGG analysis results demonstrated that 91 ERGs were mainly enriched in 4 pathways including homologous recombination, cell cycle, lysine degradation and fanconi anemia (Figure 2C, 2D).

### Identification of survival-related differentially expressed ERGs

**Figure 3:**
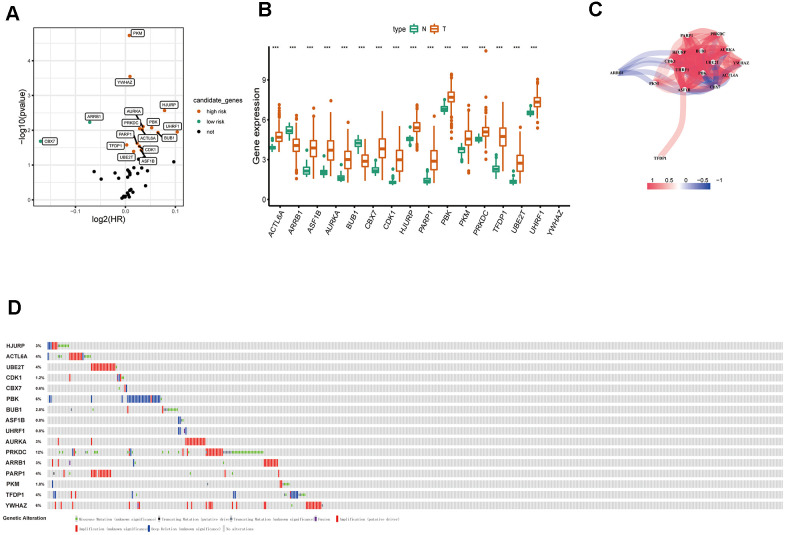
**Identification of survival-related differentially expressed ERGs.** (**A**) Volcano plot showing survival-related ERGs. P values <0.05 are considered to be statistically significant. (**B**) The expression of epigenetic-related prognostic genes between LUAD and normal tissues in TCGA database. (**C**) The correlation network of candidate genes. The correlation coefficients are represented by different colors. (**D**) Mutation frequency of candidate genes based on the cBioportal database.

By using univariate Cox regression analysis, we identified 16 ERGs that were significantly associated with prognosis in patients with LUAD (Table 1) (*P*<0.05; Figure 3A). Of the 16 genes, 14 were identified as risk factors and two were identified as protective factors. UHRF1, HJURP, BUB1, PBK, AURKA, ACTL6A, ASF1B, PRKDC, CDK1, PARP1, UBE2T, YWHAZ, PKM, and TFDP1 were identified as risk factors (*P*<0.05; HRs, 1.0012-1.0740). Whereas ARRB1 and CBX7 were considered as protective factors (*P*<0.05; HRs, 0.9512 and 0.8885, respectively). The expression of 16 ERGs in LUAD and normal lung samples were presented in Figure 3B, and the correlation between these ERGs could be seen in Figure 3C. Due to the important prognostic value of the candidate genes, their genetic alterations were analyzed. As shown in Figure 3D, missense mutation is the most common type of mutation, and there are 10 genes with mutation rate ≥3%, among which PRKDC mutation is the most frequent (12%).

**Table 1:** Univariate and multivariate Cox regression analyses of OS in LUAD patients.

**Genes**	**Univariate analysis**	**P**	**Multivariate analysis**	**P**	**Coef**
	**HR (95% CI)**		**HR (95% CI)**		
ARRB1	0.9512(0.9180-0.9857)	0.0059	0.9636(0.9318-0.9965)	0.0306	-0.0371
PARP1	1.0158(1.0021-1.0299)	0.0238	1.0156(1.0020-1.0294)	0.0251	0.0155
PKM	1.0049(1.0027-1.0072)	<0.0001	1.0043(1.0018-1.0068)	0.0007	0.0043
TFDP1	1.0012(1.0001-1.0023)	0.0266	1.0011(0.9999-1.0023)	0.0665	0.0011
YWHAZ	1.0058(1.0027-1.0090)	0.0002	1.0045(1.0021-1.0078)	0.0070	0.0045
HJURP	1.0553(1.0188-1.0930)	0.0027			
ACTL6A	1.0231(1.0056-1.0409)	0.0096			
UBE2T	1.0109(1.0000-1.0215)	0.0409			
CDK1	1.0190(1.0019-1.0363)	0.0289			
CBX7	0.8885(0.8037-0.9822)	0.0209			
PBK	1.0366(1.0092-1.0647)	0.0086			
BUB1	1.0457(1.0010-1.0826)	0.0117			
ASF1B	1.0216(1.0012-1.0423)	0.0375			
UHRF1	1.0740(1.0163-1.1350)	0.0113			
AURKA	1.0243(1.0063-1.0426)	0.0079			
PRKDC	1.0194(1.0048-1.0342)	0.0091			

### Development of epigenetic-related signature

**Figure 4:**
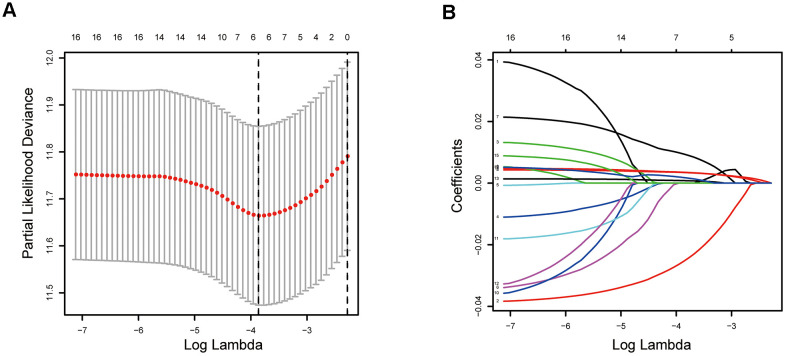
**Establishment of epigenetic-related prognostic signature.** (**A**) Screening of optimal parameter (lambda) at which the vertical lines were drawn. (**B**) Lasso coefficient profiles of these ERGs with non-zero coefficients determined by the optimal lambda.

To eliminate highly correlated ERGs and prevent overfitting of the signature, the Lasso regression analysis was performed (Figure 4A, 4B). Finally, five ERGs were confirmed by multivariate Cox regression analysis and applied to develop an epigenetic-related prognostic signature (Table 1). The signature was established to assess the prognosis of each LUAD patient as follows:

Risk score = (-0.03706×expression level of ARRB1) + (0.015474×expression level of PARP1) + (0.004509×expression level of YWHAZ) + (0.001132×expression level of TFDP1) + (0.004303×expression level of PKM).

**Figure 5:**
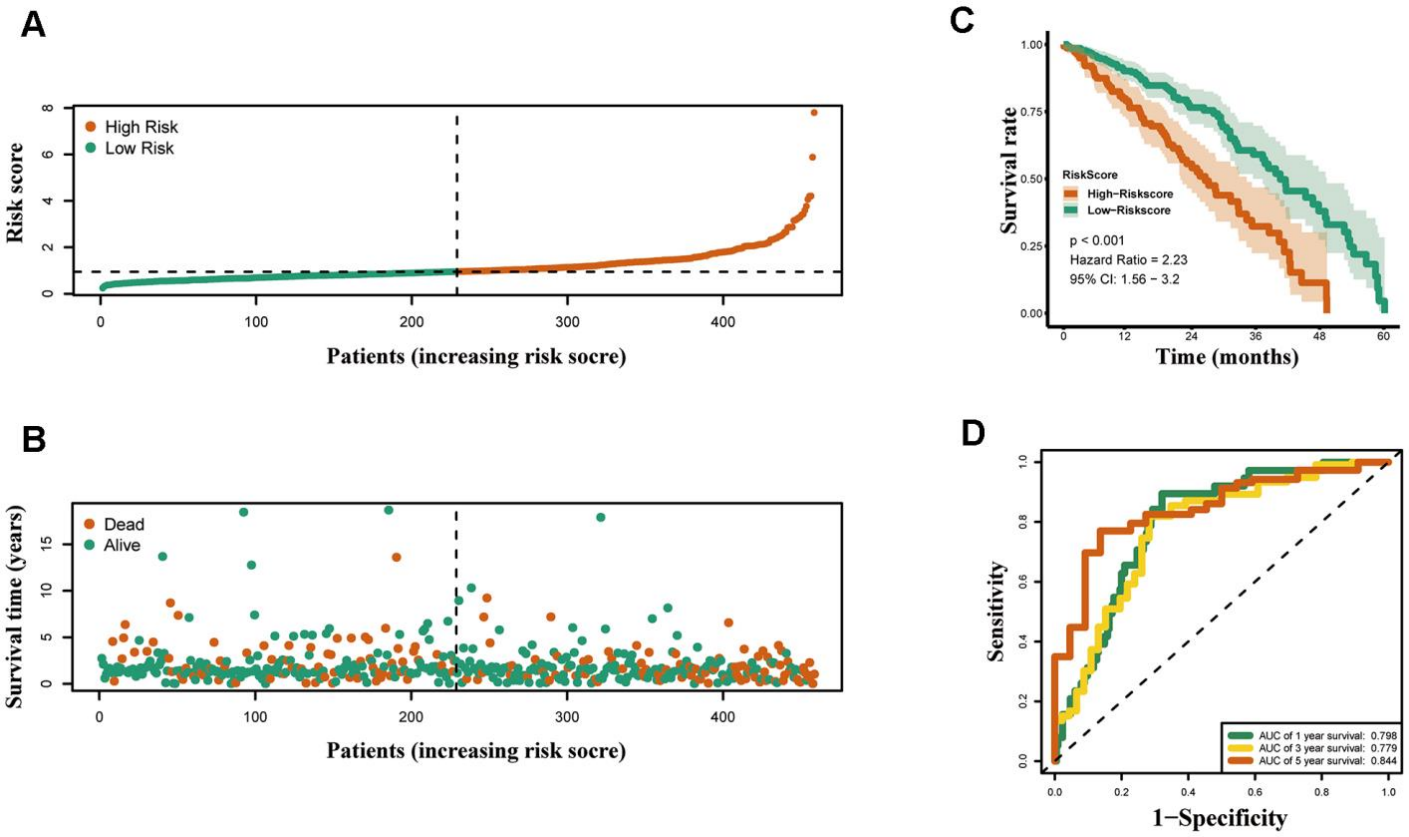
**Construction of the epigenetic-based prognostic risk signature in the TCGA cohort.** (**A**) The risk score distribution of LUAD patients. (**B**) Survival status and duration of patients. (**C**) Survival curves for the low risk and high risk groups. (**D**) Time-independent receiver operating characteristic (ROC) analysis of risk scores for prediction the overall survival in the TCGA set.

According to above formula, the risk scores of 426 LUAD patients were calculated, and patients were clustered into high and low risk groups by median risk score. The risk score (Figure 5A) and survival status (Figure 5B) of 426 LUAD patients are presented. As shown in Figure 5C, patients in the high risk group had significantly poorer OS compared to those in the low risk group. The prognostic signature exhibited good predictive performance and accuracy for predicting the 1-, 3- and 5-year OS (Figure 5D).

### Relationships between risk score and clinicopathological factors

**Figure 6:**
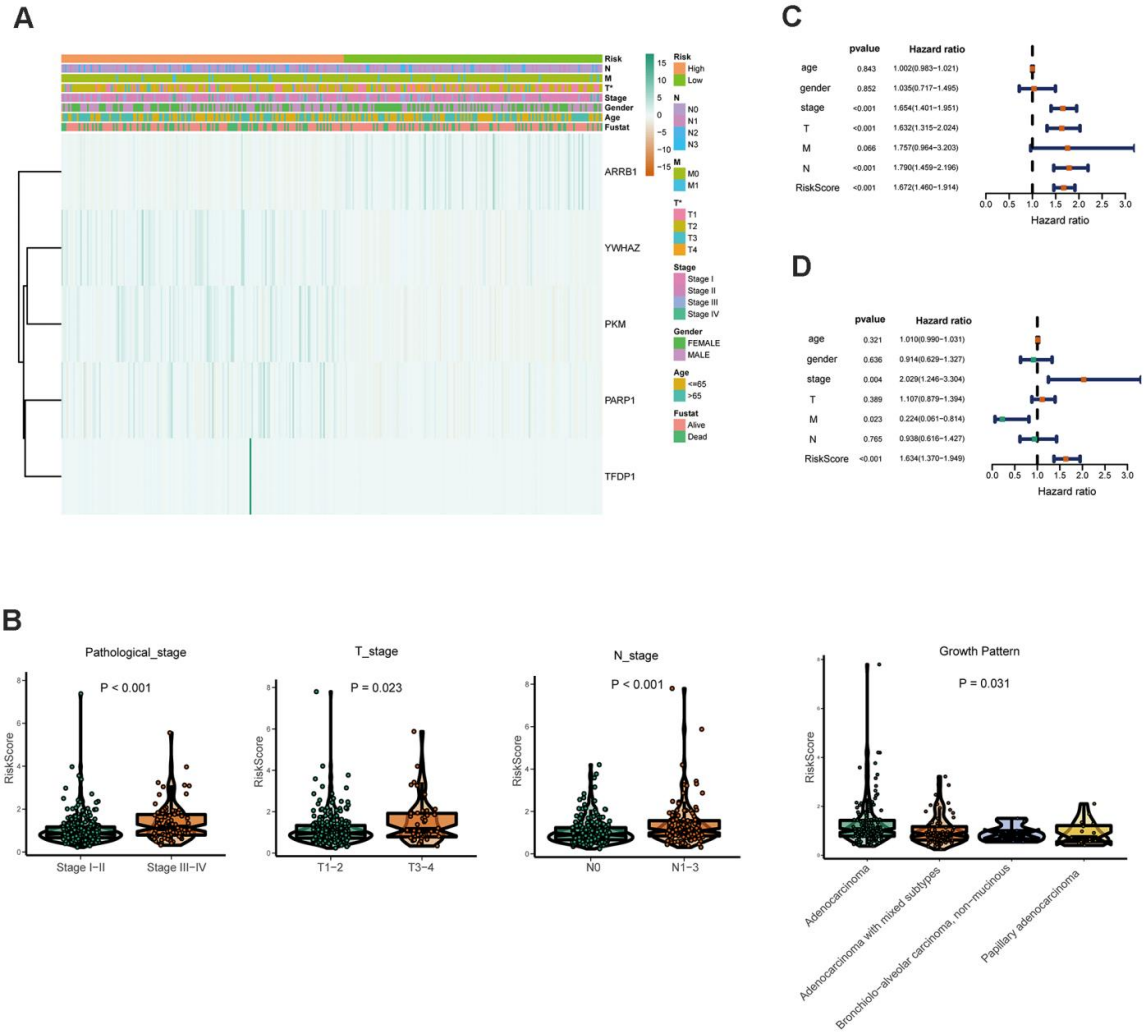
**Relationships between the risk assessment score expression and clinicopathological factors in LUAD.** (**A**) Heatmap of the epigenetic-related genes expression in the high and low risk groups and the clinicopathologic differences between the two groups. (**B**) Boxplots show the risk assessment score of patients with different pathological stage, T_stage, N_ stage and growth pattern. Univariate (**C**) and multivariate (**D**) independent prognostic analysis of independent risk factors for overall survival (OS) in patients with LUAD.

Furthermore, the expression of the five crucial genes and clinicopathological factors in high and low risk groups are presented in Figure 6A. The results indicated that with reduction of the risk score, ARRB1 was gradually increased, while YWHAZ, PKM, PARP1, and TFDP1 were gradually decreased. Significant differences were found for the pathologic T stage between the high and low risk groups. Moreover, a higher risk score was associated with higher pathological stage, N-stage and some histological growth patterns, including adenocarcinoma, adenocarcinoma with mixed subtypes, bronchioloalveolar carcinoma, non-mucinous, and papillary adenocarcinoma (Figure 6B).

Next, Univariate Cox analysis data demonstrated that pathologic stage, T stage, N stage and the risk score were all associated with OS (Figure 6C). Multivariate analysis data demonstrated that stage, M and the risk score *(P*<0.001) could be used as an independent prognostic factors (Figure 6D). Moreover, YWHAZ is significantly correlated with pathological stage, PKM is significantly correlated with pathological stage and N stage *(P* < 0.05, Table 2). Then the relationship between the risk score and clinicopathological factors was assessed. The results indicated that patients with high risk scores were positively correlated with advanced tumor stage, higher T stage, and higher N stage (*P* < 0.05, Table 2).

**Table 2:** Relationship between the expression of epigenetic-related genes and clinical characteristics.

**Gene symbol**	**Age (≥65/<65)**	**Gender (male/female)**	**Pathological stage (IV-III/ I–II)**	**T stage (T3–T4/T1–T2)**	**N stage (N2–3/N0-N1)**	**M stage (M1/ M0)**
	**t(P)**	**t(P)**	**t(P)**	**t(P)**	**t(P)**	**t(P)**
ARRB1	-1.513(0.131)	-1.635(0.103)	1.487(0.139)	0.507(0.614)	1.17(0.243)	0.395(0.697)
PARP1	0.934(0.351)	-0.761(0.447)	-0.344(0.732)	-0.269(0.789)	-0.628(0.531)	-1.23(0.233)
YWHAZ	0.369(0.713)	-1.728(0.085)	**-2.189(0.031)**	-1.72(0.092)	-1.302(0.194)	-0.694(0.495)
TFDP1	1.113(0.267)	-1.077(0.283)	-1.035(0.304)	1.155(0.249)	-1.083(0.281)	-1.029(0.316)
PKM	-1.609(0.109)	-0.663(0.508)	**-2.865(0.005)**	-1.936(0.059)	**-3.006(0.003)**	-1.352(0.191)
riskScore	0.612(0.541)	-1.205(0.229)	**-3.031(0.003)**	**-2.092(0.042)**	**-2.704(0.008)**	-1.997(0.060)

Note: t: t value of student's t test; P: P-value of student's t test.

### Validating the performance of the epigenetic-related signature

**Figure 7:**
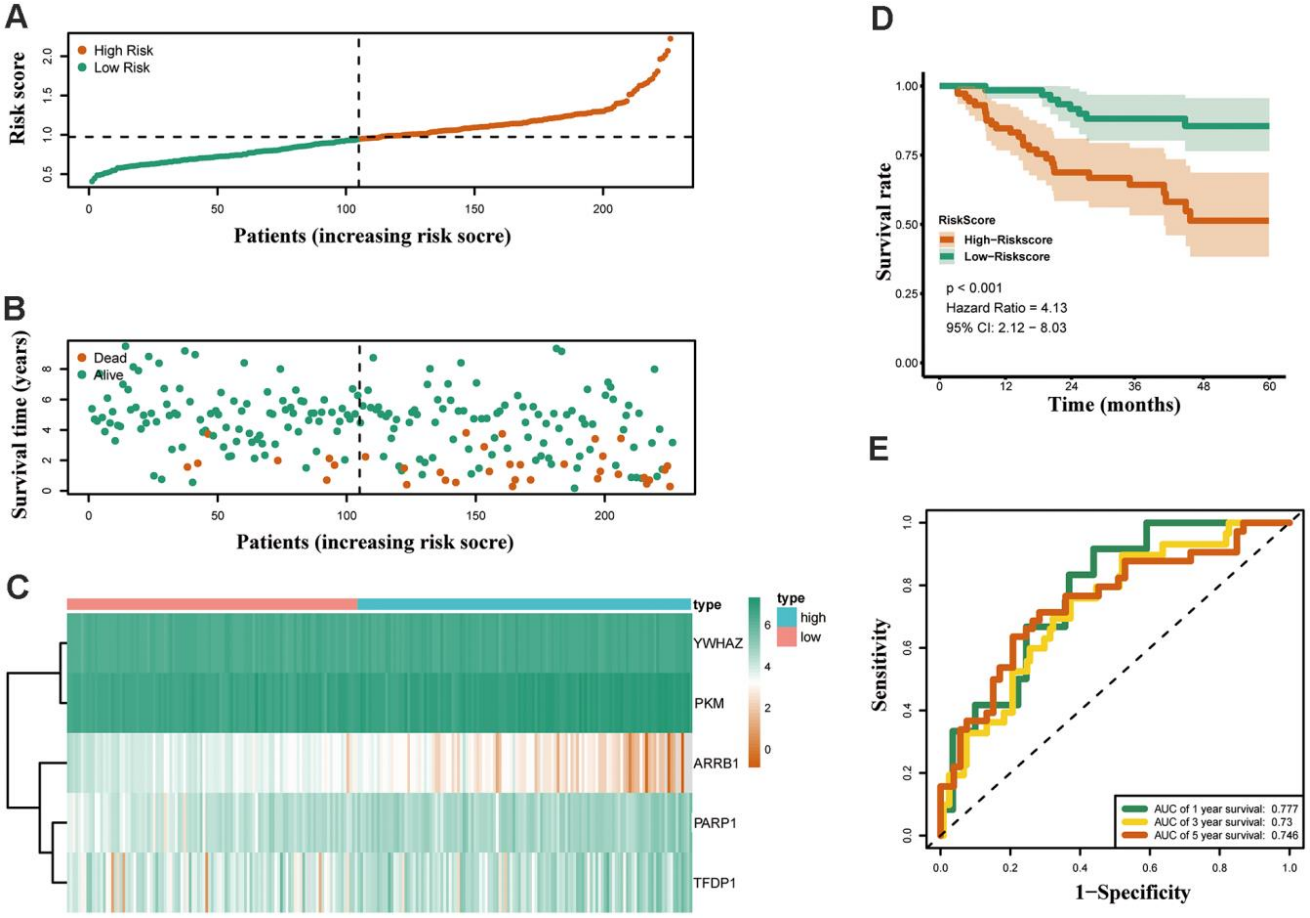
**Validation of the epigenetic-based prognostic risk signature in the GSE31210 cohort.** (**A**) The risk score distribution of LUAD patients. (**B**) Survival status and duration of patients. (**C**) Heatmap of the epigenetic-related genes expression. (**D**) Survival curves for the low risk and high risk groups. (**E**) Time-independent receiver operating characteristic (ROC) analysis of risk scores for predicting the overall survival in the GSE31210 set.

**Figure 8:**
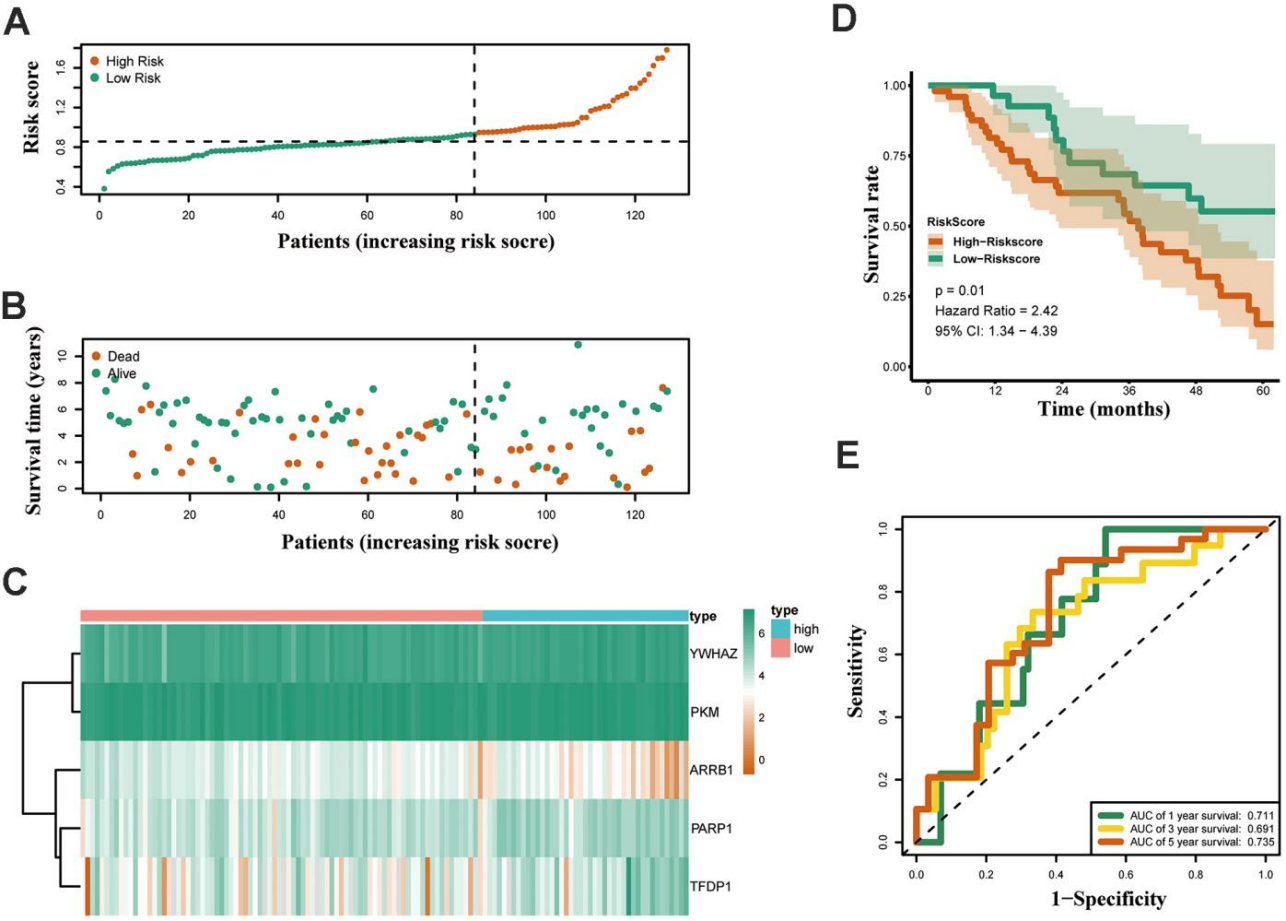
**Validation of the epigenetic-based prognostic risk signature in the GSE50081 cohort.** (**A**) The risk score distribution of LUAD patients. (**B**) Survival status and duration of patients. (**C**) Heatmap of the epigenetic-related genes expression. (**D**) Survival curves for the low risk and high risk groups. (**E**) Time-independent receiver operating characteristic (ROC) analysis of risk scores for predicting the overall survival in the GSE50081 set.

**Figure 9:**
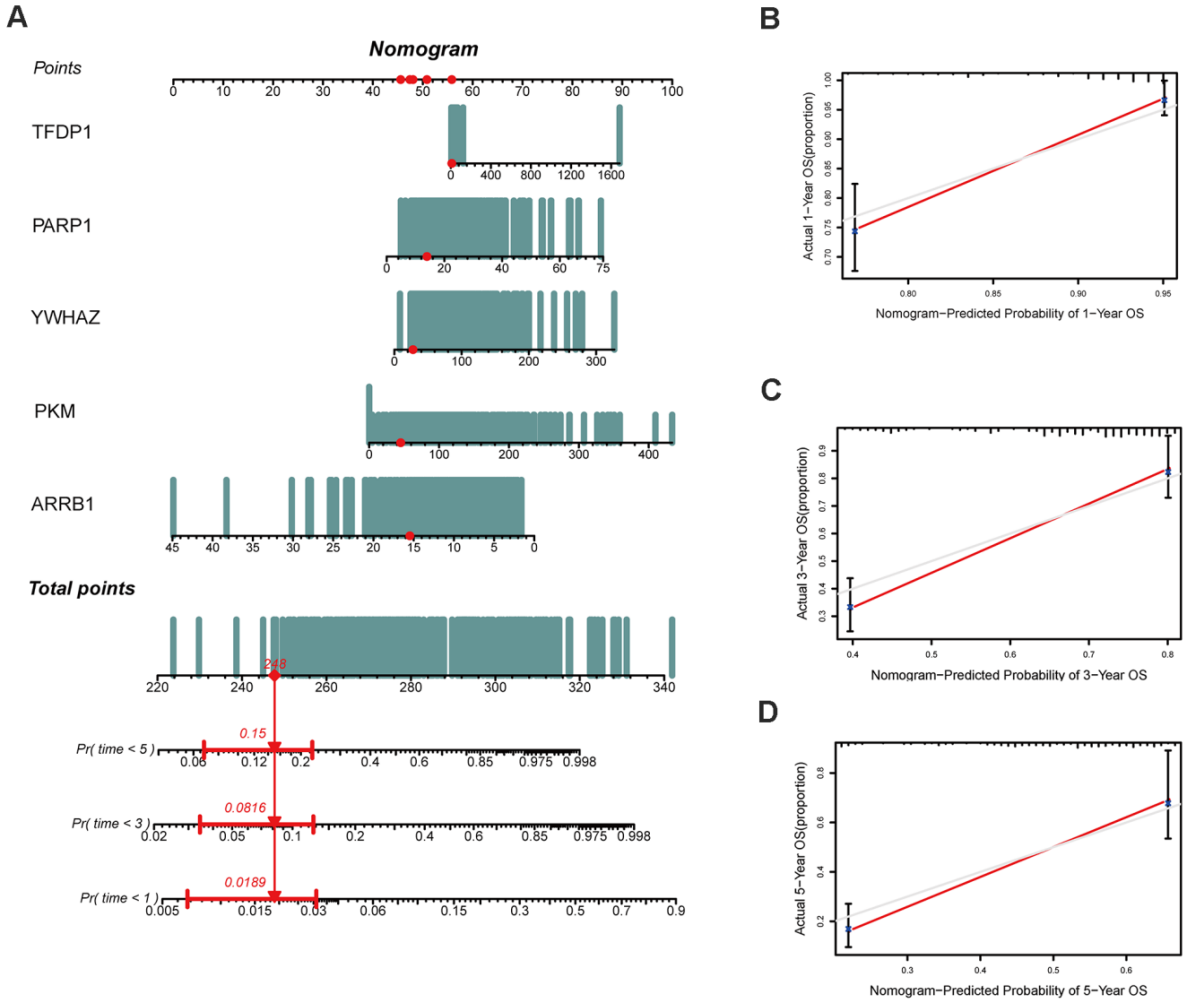
**Construction of a nomogram based on the epigenetic-related signature in the TCGA cohort.** (**A**) The nomogram based on the signature in LUAD patients at 1, 3, and 5 years. (**B**–**D**) Calibration curves of nomogram for the signature at 1, 3, and 5 years.

Both GSE31210 and GSE50081 datasets, including 174 and 127 LUAD samples, respectively, were used for validation. Consistent with our above results, the Kaplan-Meier curve showed that patients in the high risk group had a worse prognosis (Figures 7D, 8D). The risk score (Figures 7A, 8A), survival status (Figures 7B, 8B) and gene expression heatmap (Figures 7C, 8C) of five ERGs are shown, respectively. The ROC curves were built to verify the predicted capability of five ERGs, and the AUC values for 1, 3 and 5-year survival were 0.777, 0.73, 0.746 (GSE31210, Figure 7E) and 0.711, 0.691, 0.735 (GSE50081, Figure 8E), respectively. A nomogram for predicting OS was established based on the five ERGs (Figure 9A). Moreover, the 1, 3, and 5-year OS predicted by our nomogram were remarkable consistent with the actual observed survival (Figure 9B–9D).

### Exploring the expression and prognostic of crucial ERGs

**Figure 10:**
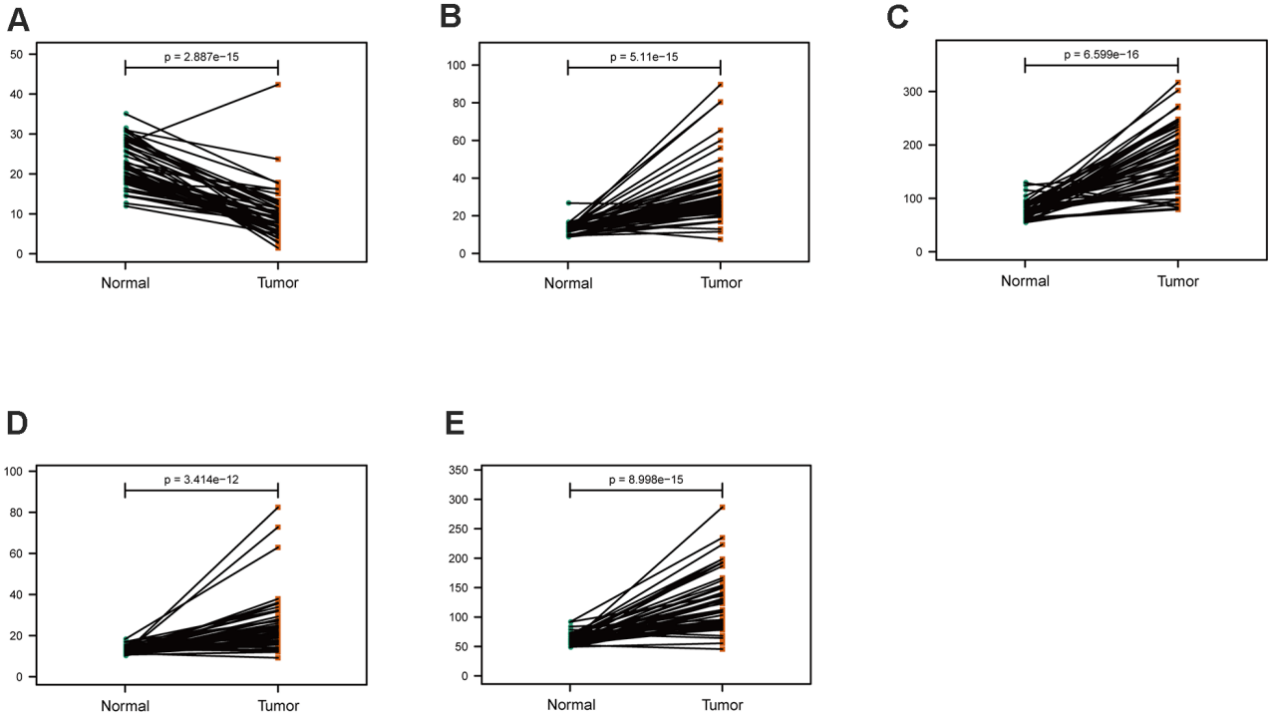
**Comparison of the hub genes mRNA levels in paired adjacent normal tissues and LUAD tissues from the TCGA cohort.** (**A**) ARRB1, (**B**) PARP1, (**C**) PKM, (**D**) TFDP1, (**E**) YWHAZ.

**Figure 11:**
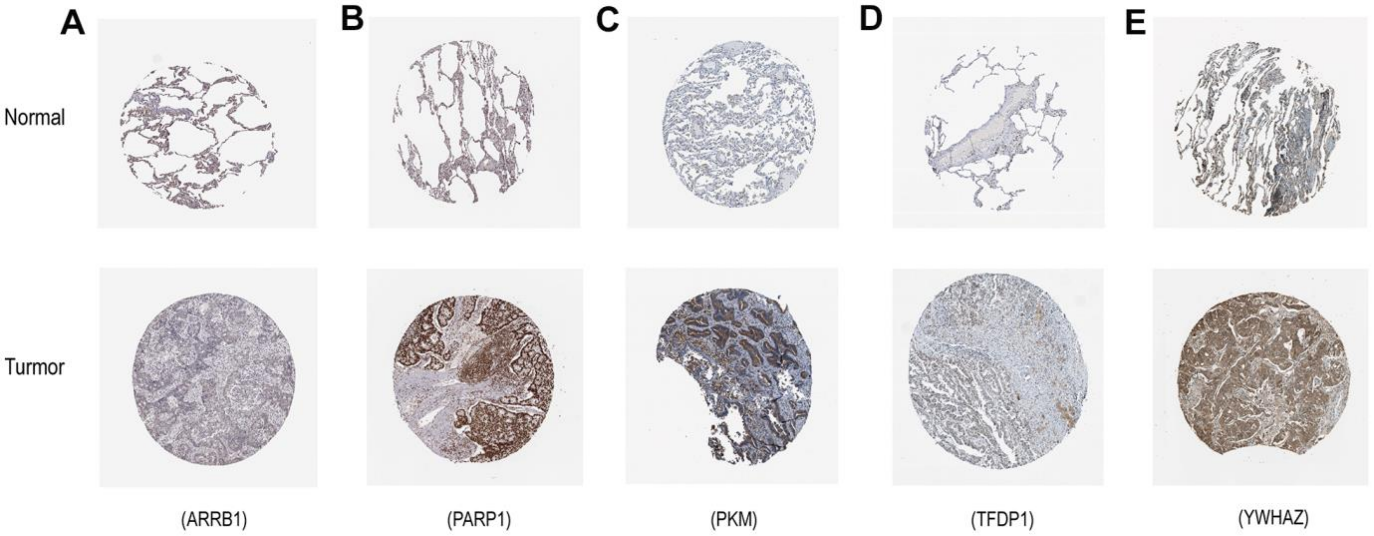
**Verification of hub ERGs expression in LUAD and normal lung tissue using the HPA database.** (**A**) ARRB1, (**B**) PARP1, (**C**) PKM, (**D**) TFDP1, (**E**) YWHAZ.

**Figure 12:**
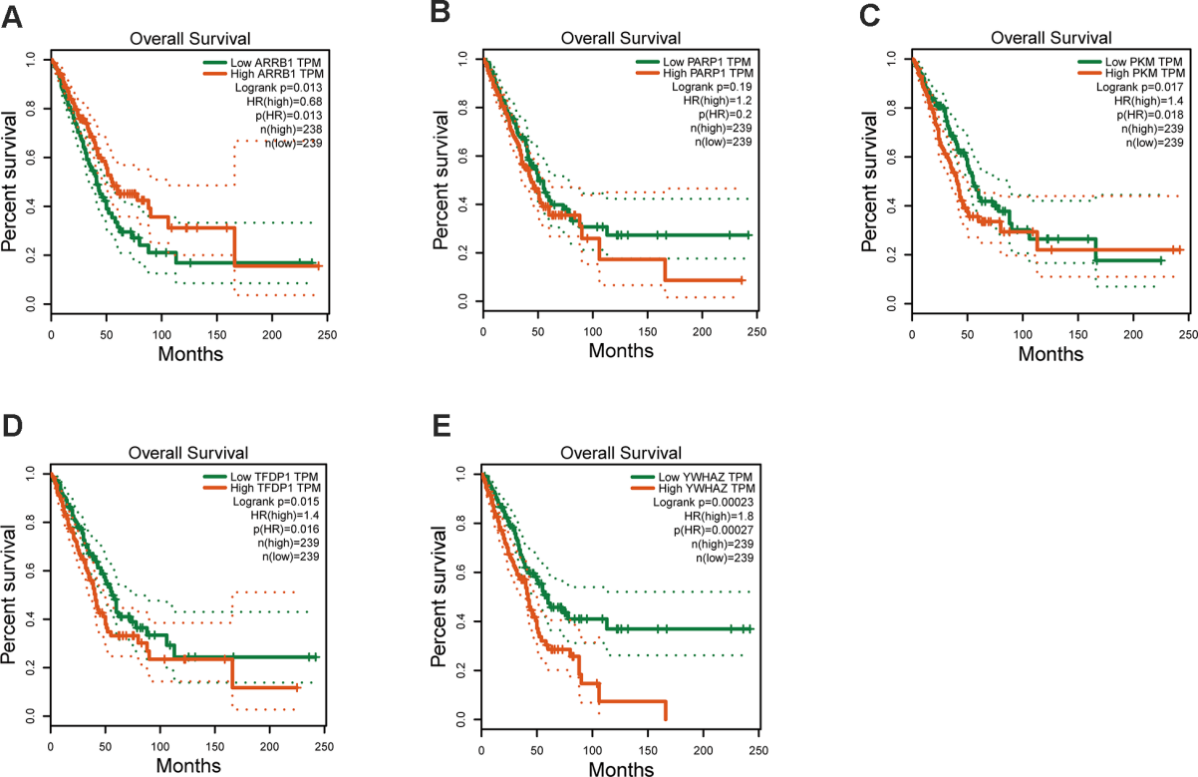
**Kaplan–Meier survival analysis is performed on the hub genes.** (**A**) ARRB1, (**B**) PARP1, (**C**) PKM, (**D**) TFDP1, (**E**) YWHAZ.

The mRNA expression of the five hub genes in LUAD were analyzed by using the TCGA database. We confirmed that the mRNA expression of PARP1, PKM, TFDP1, and YWHAZ in LUAD tissues were all increased, while ARRB1 was decreased (Figure 10). Moreover, we explored the protein expression of the five hub genes. The results indicated that the protein of PARP1, PKM, TFDP1, and YWHAZ were increased in LUAD tissues, which were in line with their mRNA expression levels (Figure 11). Furthermore, we found that the expression of the three high risk genes (PKM, TFDP1, and YWHAZ) were negatively associated with the prognosis in LUAD, while low risk gene ARRB1 was positive correlation with the prognosis by using the GEPIA database (Figure 12).

### Identification of potential candidate drugs targeting the signature

**Figure 13:**
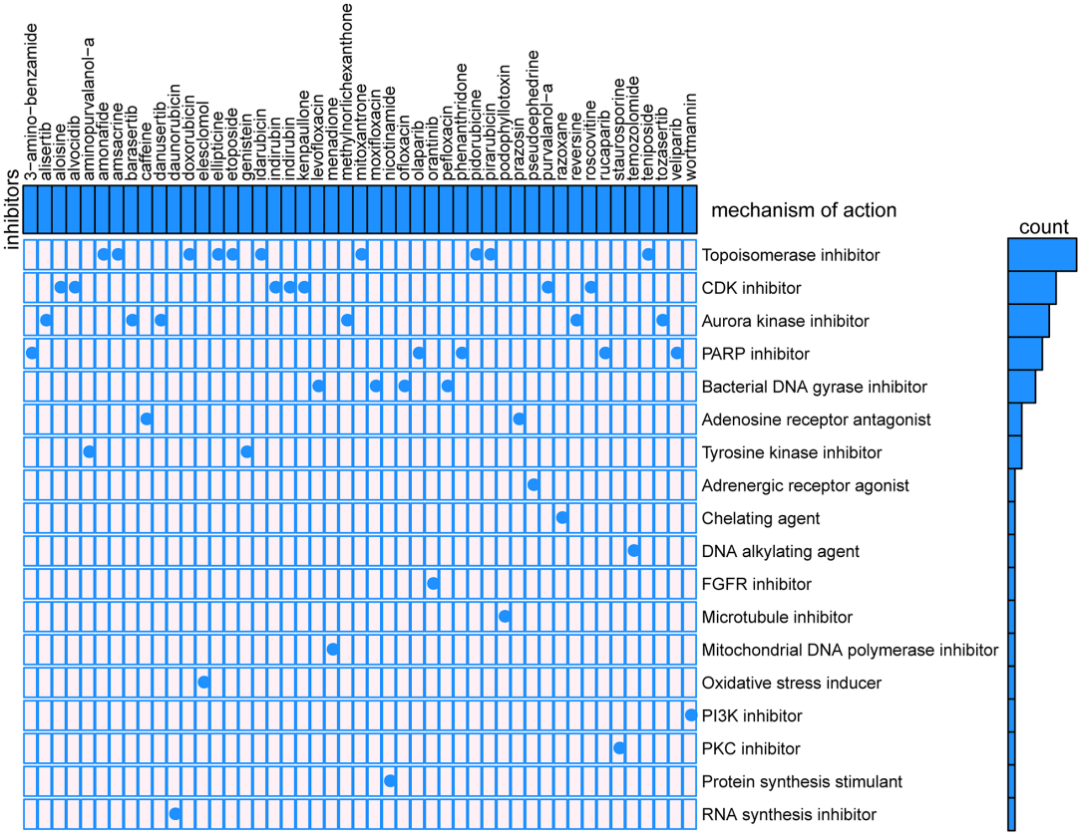
Results of CMap analysis.

CMap mode-of-action (MoA) analysis found that 47 compounds could be candidate drugs targeting the signature and the drugs shared 18 kinds of mechanisms (Figure 13), ten drugs shared the MoA of topoisomerase inhibitor (amonafide, amsacrine, doxorubicin, ellipticine, etoposide, idarubicin, mitoxantrone, pidorubicine, pirarubicin, teniposide), seven drugs shared the MoA of CDK inhibitor (aloisine, alvocidib, indirubin, indirubin, kenpaullone, purvalanol-a, roscovitine), six drugs shared the MoA of aurora kinase inhibitor (alisertib, barasertib, danusertib, methylnorlichexanthone, reversine, tozasertib), and five drugs shared the MoA of PARP inhibitor (olaparib, phenanthridone, rucaparib, veliparib, 3-amino-benzamide).

## DISCUSSION

Lung adenocarcinoma, a molecularly complex and heterogeneous disease, remains the most common causes of cancer-associated deaths worldwide [1, 20]. Recently, the development of drugs targeting on EGFR [5] and ALK [7] has promoted the treatment of some patients with LUAD, however, the highly gene mutation heterogeneous of LUAD has limited the benefits to a small number of patients. Notably, there is growing evidence that epigenetic modifications are frequently reversible, and may serve as attractive targets for lung cancer therapy [21, 22]. Therefore, it is particularly important to identify suitable epigenetic markers for LUAD diagnosis and prognosis, that can provide valuable support in decision making when considering treatment options.

In this study, we developed a novel and meaningful epigenetic-related prognostic signature (ARRB1, YWHAZ, PKM, PARP1, and TFDP1) for LUAD patients and validated them in two independent datasets from GEO. According our risk score system, LUAD patients were stratified into high and low risk groups, and patients in the high risk group had a worse prognosis. Then, we confirmed that each of the five signatures could be an independent prognostic factor. A new nomogram was constructed based on the five hub genes, which can predict the OS of every LUAD patients. Interestingly, the expression of three high risk genes (PKM, TFDP1, and YWHAZ) had an enormous implication in the prognosis of LUAD patients. Finally, we identified 47 compounds that could serve as candidate targeted chemotherapeutic drugs to the five ERGs for LUAD patients. These drugs included the topoisomerase inhibitor, CDK inhibitor, aurora kinase inhibitor, PARP inhibitor, and so on.

The epigenetic factors included in our signature have been previously demonstrated to be closely related to the development and progression of lung cancer. ARRB1 (also known as β-arrestin-1), a multifunctional adaptor, was initially discovered to promote internalization and desensitization of G protein-coupled receptors (GPCRs) [23, 24]. A study by Pillai et al. revealed that ARRB1 promoted the expression of mesenchymal genes through mediation of the E2F1 transcription factor in non-small cell lung carcinoma cell lines (NSCLC) [25]. Likewise, Shen et al. reported that ARRB1 could enhance the chemo-sensitivity of lung cancer through the mediation of DNA damage response [26]. Our results suggested that ARRB1 may be a putative tumor suppressor gene in LUAD. The M2 isoform of pyruvate kinase (PKM2) (also named PKM, PK3, THBP1), an essential enzyme involved in glycolysis, is known to mediate the conversion of glucose to lactate in cancer cells under normoxic conditions [27, 28]. Jing Li reported that phosphorylation of PKM2 and inactivation of STAT3 inhibited lung cancer cell proliferation [29]. Inhibitors of PKM2 could moderately decelerate tumor cell proliferation [30, 31]. This finding might be in agreement with our current results, that PKM2 is a high risk gene in the context of LUAD. PARP1 has been reported as an abundant chromatin-associated enzyme able to catalyze the transfer of ADP-ribose units from NAD to substrate proteins [32]. Alternatively, PARP1 might play a important role in the development of various cancers, including cell proliferation, apoptosis, DNA repair, and so forth [33]. Recently, the use of immune checkpoint inhibitors has shown dramatic effect in the prognosis of NSCLC. Sophie Postel-Vinay reported that PARP inhibition enhanced the intrinsic immunity of tumor cells in NSCLC with deficiency in excision repair cross-complementing group 1, a gene which has crucial role in the nucleotide excision repair [34]. They also suggested that PARP inhibition, which did not cause severe bone marrow toxicity, might represent an interesting alternative or complement to platinum-based chemotherapy in combination with anti–PD-(L)1 agents in NSCLC. Transcription factor Dp-1 (TFDP1) is a key player of cell cycle regulation, it is a predominant protein that binds to E2F, another vital transcription protein that participates in the control of cell cycle and action of tumor suppressor proteins. TFDP1 can be candidate master regulators contributing to follicular lymphoma progression [35]. Knockdown of TFDP1 reduced both PITX1 promoter activity and mRNA transcription which caused patients suffering of knee/hip osteoarthritis [36]. Wang et al. revealed that TFDP1/E2F1 transcriptional activity played an important role in NSCLC [37]. YWHAZ (also named 14-3-3ζ), acts as a major hub protein for many signal transduction pathways [38, 39]. YWHAZ was frequently shown to be upregulated in several types of cancers, and its overexpression was often correlated with unfavorable prognosis of cancer patients [40–43]. Ma et al. found that YWHAZ was a credible prognostic biomarker, and might be a therapeutic target in NSCLC [44].

Overall, in concordance with our findings, there is mounting evidence in the literature that tells about the important role of the 5 hub genes (described above) in relation to NSCLC, which further supports the use of these hub genes as prognostic genes for LUAD patients. In recent years, a great deal of knowledge has been accumulated to help identify prognostic signature of different types of cancers, one of which is NSCLC. In our study, we not only identified the five powerful ERGs that are useful as prognostic signature, also provided potential drugs for targeted therapy. Interestingly, PARP inhibitors are currently regarded as a novel class of small molecule therapeutics for lung cancer. Henning Willers and colleagues found that PARP inhibitor by controlling ROS levels upon EGFR TKI treatment, promoted the sensitivity of EGFR-mutated lung cancer to tyrosine kinase inhibitor treatment [45]. We hope our study will provide more choice for those patients not having EGFR mutation.

In the present study, there are some limitations that require mentioning. First, the three datasets are all retrospective and, hence, extrapolation based on these results may be difficult. To that end, the findings of this work should be validated, and further exploration using a larger multicenter prospective observational trial is desirable. Second, our findings have to be enriched by conducting long term *in vivo* experiments and additional *in vitro* experiments to investigate the functional role of the epigenetic factors associated with LUAD.

In summary, five ERGs (ARRB1, PARP1, PKM, TFDP1, and YWHAZ) are promising biomarkers for the diagnosis and prognosis of LUAD, which could provide valuable references to identify whether LUAD patients are at high risk. In addition, our findings may provide precise targeted chemotherapeutic drugs.

## MATERIALS AND METHODS

### Study subjects

The RNA-seq data of 497 LUAD and 54 normal lung samples were downloaded from The Cancer Genome Atlas (TCGA) database, and the RNA-seq data of 174 LUAD and 127 LUAD samples were downloaded from GSE31210 and GSE50081 dataset, respectively. A total of 720 epigenetic-related genes (ERGs) were retrieved from the EpiFactors database (http://epifactors.autosome.ru/).

### Identification and enrichment analysis of differentially expressed ERGs

Differentially expressed ERGs were obtained by using the Limma package in R software [46]. To further investigate the biological relevance of these genes, Gene Ontology (GO) and Kyoto Encyclopedia of Genes and Genomes (KEGG) pathway enrichment analyses were conducted utilizing R “GOplot” package.

### Development of the epigenetic-related signature for patients with LUAD

We collected clinical information of LUAD patients who were followed for less than 2000 days in the TCGA database. Univariate Cox regression analysis was performed by R “survival” package to identify ERGs markedly related to OS. Genetic alterations in the candidate genes were obtained from cBioPortal (https://www.cbioportal.org/). Then, a novel epigenetic-related prognostic signature was developed by Lasso and multivariate Cox regression analyses [47, 48]. The risk scores of LUAD patients were calculated according to the formula: the signature risk score = Ʃ (βi × Expi), where βi, the coefficients, represented the weight of the respective signature and Expi represented the prognostic factors expression value as previously described [49]. According to the signature with identified prognostic factors, a nomogram for predicting the probability of OS was established.

### Validating the performance of the epigenetic-related signature

To validate the performance of the signature, the GSE31210 and GSE50081 datasets were considered as the validation cohort. The risk scores for LUAD patients were calculated by using the formula. Survival and ROC curve analyses were performed as described above.

### Exploring the expression and prognostic of crucial ERGs

To explore the prognostic value and the expression of these ERGs in LUAD, survival analysis was conducted on GEPIA database (http://gepia.cancer-pku.cn), the expression of these ERGs were confirmed on Human Protein Atlas (HPA) online database (http://www.proteinatlas.org/) [50].

### Predicting candidate small molecules for LUAD patients

To predict potential drugs for LUAD patients, we utilized the Broad Institute’s Connectivity Map (CMap) to screen candidate molecule drugs as previously described [51, 49].

### Statistical analysis

In this study, all statistical analyses were conducted using Perl software (version 5.28.1) and R platform (version 3.6.1). The comparison of two paired groups was performed using the Wilcoxon rank-sum test. P value <0.05 was considered as statistically significant.
